# A personal historic perspective on the role of chloride in skeletal and cardiac muscle†

**DOI:** 10.14814/phy2.13165

**Published:** 2017-03-21

**Authors:** Otto F. Hutter

**Affiliations:** ^1^ PhysiologyUniversity of GlasgowGlasgowUnited Kingdom of Great Britain and Northern Ireland

**Keywords:** cardiac muscle, chloride, skeletal muscle

## Abstract

During the early decades of the last century, skeletal muscle was held to be impermeable to chloride ions. This theory, based on shaky grounds, was famously falsified by Boyle and Conway in 1941. Two decades later and onwards, the larger part of the resting conductance of skeletal muscle was found to be due to chloride ions, sensitive to the chemical environment, and to be time‐and‐voltage dependent. So, much of the groundwork for the physiological role of chloride ions in skeletal muscle was laid before the game‐changing discovery of chloride channels. The early history of the role of chloride in cardiac muscle, and work on the relative permeability to foreign anions of different muscles are also here covered from a personal perspective.

## Introduction

The best philosophers illumine our thoughts and guide our actions. Since Karl Popper proved that scientific knowledge advances through conjectures and refutations (Popper 1963), that insight has become the recurrent theme of numerous retrospective scientific narratives. I also have already borrowed this leitmotiv in a different context on a previous occasion (Hutter, 2006). Here, I turn to how our knowledge of the role of chloride in muscle has grown because that subject illustrates *par excellence* how scientific knowledge advances through the falsification of theories. Also, the role of chloride in muscles is a topic to which I happen to have made contributions way back in the 1950s and 1960s. And I have watched later developments with ever increasing fascination. So this will be a personal, a subjective account by an old hand in the game.

I started to study physiology in 1942, when at age 18 I took a war‐time job as a laboratory technician at the Wellcome Physiological Research Laboratories. Quite apart from the rich laboratory experience I gained there, I also received a wonderful continuing education. Many of the war‐time scientific staff at the laboratories had been University Lecturers, and they were only too willing to guide me in my studies as an external student.

The text books from which I studied were inevitably of pre‐war vintage. As regards the role of ions in muscle, the latest authoritative account was an article in *Physiological Reviews* by Fenn (1936). In it he concludes:

“This review has been presented chiefly from the point of view of the theory that the muscle membrane is impermeable to all common ions except the potassium and the hydrogen ion. This theory explains satisfactorily most of the facts”. What were the grounds which convinced a good scientist like Wallace Fenn that skeletal muscle fibers were not permeable to chloride ions?

Three, at the time seemingly valid findings, underpinned that conclusion: First, way back, Höber (1905) showed that the resting potential of a sartorius muscle – as measured between one external electrode on an injured tibial end of the muscle, and another on its intact pelvic end – that the resting potential so measured was not altered when extracellular chloride was replaced by sulphate or sucrose. If chloride were permeable, he argued, then a reversal of the chloride concentration gradient should have caused a depolarization. As we shall see later, that argument was correct, but the experimental set‐up was inadequate.

Secondly, one can point to work on model membranes done by Michaelis (1925). He showed that the ionic permeability of collodion membranes depended on the charge of molecules admixed into the collodion film. When a collodion membrane was doped so as to become negatively charged, it repelled chloride but admitted potassium. This was an influential finding, and Michaelis model seemed persuasively applicable to the skeletal muscle membrane (Mond and Amson 1928).

Finally, Fenn et al. (1934) concluded, on basis of histological measurements, that the extracellular spaces between muscle fibers can accommodate all the chloride within an anatomical muscle, implying that the muscle fibers themselves are free of chloride.

But, all this proved to be a pack of cards that was blown over by the great Irish physiologist E J Conway, born in Co Tipperary in 1897. As a schoolboy, he carried off all the prizes. And after qualifying in medicine, he joined the Physiology Department in Dublin. At first, he engaged in research on renal physiology. Then, in 1937, he and his colleagues showed that muscle was permeable to lactate. That was the first demonstration that an anion could cross the muscle membrane, but it could be waved away by supposing that the permeating species was not the lactate anion, but rather undissociated lactic acid.

Then, in 1939, in a letter to *Nature*, Conway & Boyle put forward a new model for the muscle membrane in the following words: “The membrane considered, is one of special type with cation *and* anion pores, permeable to potassium but not to the larger sodium ions, and permeable at the same time to small anions of the type of chloride …”

Here (omitted) is a pictorial representation of that model. The pores in the membrane are shown large enough to admit the relatively small K and Cl ions. But the membrane pores are too small for the internal macromolecular anions and for the more heavily hydrated external Na ions; and for present purposes, it does not matter whether Na is really impermeable or only effectively so. This model is sometimes called a Double Donnan Equilibrium, after another great Irish scientist F G Donnan.

Now as is well known, according to the rules of the Donnan Equilibrium (Donnan, 1911) the permeable ions will distribute themselves between two compartments so that the concentration gradients of the permeant cations and anions are identical, as there can be only one membrane potential; or to put it another way for the present case, so that the product of the outside concentrations [K_o_] x [Cl_o_] is equal to the product of the inside concentrations [K_i_] x [Cl_i_]. So, following in Donnan's footsteps, Boyle and Conway argued that because the inside concentration of potassium is so high, the inside concentration of chloride may well be vanishingly low.

Two major papers from Conway's laboratory followed. In the first, Boyle et al. (1941) arrived at a lower estimate for the extracellular space, than that previously estimated by Fenn et al. (1934). This allowed for a finite but low intracellular Cl^−^ concentration in accordance with a Donnan equilibrium. In the second, famous paper, Boyle and Conway (1941) demonstrated – in a variety of elegant ways mostly involving measurements of muscle volume changes – the ability of chloride to enter muscle.

As to the mechanism by which both potassium and chloride ions may cross the membrane, Boyle and Conway (1941) surmised: “The similarity of anion and cation diameters for diffusion through the membrane suggests the view that the same molecular pore exists for both and that this is probably not charged within the membrane”. Essentially, this was still the “pore‐sieve” model of ion permeation first put forward by Ostwald at the end of the 19th century. So, as regards the mechanism of ion permeation, no great conceptual advance was made during the first part of the 20th century. But importantly, Conway overturned an ill‐founded dogma which had held sway for too long.

Interestingly, Conway and Boyle's 1939 letter to *Nature* appeared in the same issue as Hodgkin & Huxley's famous letter in which they first demonstrated the overshoot of the squid nerve action potential. In fact, these two epoch defining letters immediately followed one another. But as it happened, in the immediate post‐war years, it was the role of Na and K in the generation of the action potential which claimed center stage, while the role of Cl in excitable cells remained a backwater for some more years.

It was into that backwater that I chanced to put my paddle to give it a bit of a stir. It happened this way. In the summer of 1955, when I had returned to University College London after two fruitful years in Stephen Kuffler's Laboratory in Baltimore, I was allotted a young Pakistani medical graduate named S M Padsha as my first PhD student. Looking for a promising problem, I seized upon work then just done at UCL by Hill and McPherson (1954). They had shown that replacement of chloride by nitrate prolonged the active state of muscle. Moreover, the speedy onset of that effect pointed to an action of nitrate on the surface membrane.

So, we examined what nitrate might do to the electrical properties of the muscle membrane. We soon found that replacement of chloride by nitrate doubled the muscle membrane resistance. And we were just in time to demonstrate this effect at the March 1956 meeting of the Physiological Society at UCL under the title: “Effect of nitrate on the electrotonic potential of skeletal muscle”. In those grand old days, the Demonstrations were regarded as the most important part of the meeting, a practice that has since sadly gone overboard.

The most obvious interpretation of our finding was that chloride normally contributes at least one‐half of the muscle membrane conductance. But, this met with some skepticism because to that point in time no one had shown that substitution of chloride by a less permeable anion had a major effect on the membrane potential.

Now, Alan Hodgkin always attended the UCL meeting, so he must have been aware of our demonstration, but I cannot remember whether he made any comment. At all events, he obviously savvied that if the muscle membrane were indeed really highly permeable to chloride ions, as our demonstration suggested, then external chloride would need to be removed so rapidly that the internal concentration of chloride cannot fall at the same time. And at the next UCL Meeting, in March 1957, Hodgkin & Horowitz described experiments on single muscle fibers that had been loaded with chloride by equilibration in a solution of high KCl product. If such a chloride rich fiber is then suddenly exposed to an external solution low in chloride so as to reverse the chloride concentration gradient effectively–‐ as Höber never could do – the membrane potential turns inside positive by as much as +70 mV, showing that the membrane potential can be dominated by chloride. At the same meeting, I introduced S M Padsha (1957) to communicate the effects of an extended series of foreign anions on muscle membrane resistance.

The full papers from the two teams were published in 1949: ours appeared in volume 146. In it we concluded: “…that chloride ions normally contribute the major share of the membrane conductance”*,* the justification being, that chloride replacement by iodide – the most effective anion we tested – caused as much as a 2.4‐fold increase in membrane resistance, equivalent to a fall in membrane conductance to 0.4 of its normal value.

The paper from the Cambridge team appeared in volume 148. Much of this informative paper dealt with the rectifying property of the potassium conductance. This meant that the relative contributions of the potassium and chloride conductances varied according to the experimental conditions. For muscle equilibrated in Ringer's solution, Hodgkin and Horowicz (1959) found the transport number for chloride to be 0.6, whereas that for potassium to be about 0.3. Gratifyingly, Hodgkin gave space to acknowledge and discuss our similar results; but inevitably our paper, though original and informative, remained overshadowed by the greater refinement of Hodkgin & Horowicz's use of single muscle fibers and by Hodgkin's consummate analysis of the results the single fibers yielded.

Anyhow, my first PhD student Padsha duly gained his PhD at the hands of AV Hill. My next PhD student was a conspicuously talented medical student, who had taken the intercalated BSc Physiology course and who had acquired a taste for more physiology. His name was Denis Noble. Denis joined me in September 1958. By then Harris (1958), also at UCL, had shown that the efflux of isotopic Chloride^36^ from frog muscle was slowed by nitrate and iodide. This interaction between the ions of the lyotropic series prompted us to study also the effect of replacing chloride with a noninterfering impermeable anion.

At this point, Paul Fatt came up with the suggestion that methylsulphate might do the trick, and this proved to be the case. So, Denis and I set about to compare the membrane conductance of muscle in Ringer's solution with that of muscle made chloride‐free by use of methylsulphate. Our paper appeared in April 1960. Our main conclusions were:

(1) Chloride normally contributes 68% to the resting membrane conductance, in confirmation with our earlier results with iodide. (2) The potassium conductance – that is the conductance left after the total removal of chloride – rectifies inwardly. (3) The chloride conductance – as derived by subtracting the chloride conductance from the total membrane conductance – showed outward rectification in accordance with the Constant Field Theory.

All these findings agreed with the conclusions at which Hodgkin & Horowicz had arrived at by different experimental approach. So, by the beginning of the 1960s the magnitude of the muscle chloride conductance was firmly established. But the contemporary view remained that chloride was passively distributed (Adrian 1961) and that chloride ions crossed the membrane by electro‐diffusion through an aqueous channel subject to a linear potential drop. As will become apparent, this was still a somewhat simplistic picture.

Meanwhile, Denis Noble and I continued our collaboration by examining also the role of chloride in cardiac muscle. In the course of that work, about which more later, I was offered an appointment to the scientific staff at the National Institute for Medical Research. Much, much later I learned that I owed that appointment to a recommendation by Alan Hodgkin.

In moving to Mill Hill, I was allowed to bring with me a new postgraduate student from the 1961 crop of Physiology graduates. I chose a Miss Anne Brooks, soon to become Mrs Anne Warner. I chose her mainly because of her role in the students' Operatic Society. Not as a singer, I hasten to add, rather as stage lightening technician. I argued that a girl who can handle a lot of switches probably had the makings of good electrophysiologist. And so it eventually turned out.

Once installed at Mill Hill, we set about to study the effects of pH on the chloride conductance of skeletal muscle. Remembering the work of Michaelis (1925), I expected that protonation of a membrane would make it more permeable to anions. So, imagine my surprise when we found exactly the opposite: acidification of the external solution greatly reduced the chloride conductance, whereas raising the pH increased it to about twice its normal value. Eventually, we published a series of three papers (Hutter and Warner 1967a,b,c).

The first described the surprising pH sensitivity of the chloride conductance. The second paper nailed down this effect by showing that the efflux of Cl^36^ was similarly altered by pH changes. And in the third paper, we showed that low concentrations of the metal cations Cu^2+^ or Zn^2+^ or UO^2+^ also greatly reduce the chloride conductance.

In retrospect, we should have divided the first paper because it contained a separate new finding. We found that when a muscle is left in an acid solution for some time chloride accumulates in the fiber. This pointed to the existence of an inward chloride transport system which is normally masked by the high chloride conductance. This was in fact a correctly interpreted early demonstration of the existence of active chloride transport in muscle of which more later.

Two further papers followed before I and Anne Warner left the National Institute to go our separate ways. In the first, Hutter and Warner (1972), we studied the current–voltage relationship of the chloride conductance at different pH. As before, the relationship at near‐neutral pH resembled the predictions of constant field theory. But in acid and alkaline solutions, it departed from the predictions of the constant field theory in different ways. Moreover, we found indications that the chloride conductance was time dependent in different ways depending on the external pH. These indications were then confirmed by Anne Warner in a nice last paper (Warner 1972) in which she employed the three electrode voltage clamp technique invented by Adrian et al. (1970). Whether bringing up children in a family, or young colleagues in the lab, you have to give them the opportunity eventually to do their own thing.

So, here is what we knew about chloride and skeletal muscle by the end of the 1960s. (1) Under normal conditions, chloride accounts for the larger part of the resting membrane conductance (Hutter and Padsha 1956, 1959; Hodgkin and Horowicz 1957, 1959; Hutter and Noble 1960) (2) The chloride conductance is labile, that is, sensitive to pH and metallic cations (Hutter and Warner 1967a,b,c) (3) The full range of behavior of the chloride conductance is more complex than the predictions of the Constant Field Theory (Hutter and Warner 1972; Warner 1972) (4) Under conditions of low chloride permeability, inward transport can raise the intracellular concentration of chloride to above the level expected for a purely passive distribution (Hutter and Warner 1967a) (5)The rank order of anion permeability in skeletal muscle at normal pH is Cl> Br >NO_3_ > I >SCN (Hutter and Padsha^1959^), but at pH 5.0 NO_3_ is more permeable than Cl (Hutter and Warner 1967d).

Now before dealing with later work on skeletal muscle, let me introduce some work on cardiac and crustacean muscle. When I came back to UCL, I brought with me some experience in cardiac electrophysiology thanks to a collaboration with Wolfgang Trautwein (Hutter and Trautwein 1956). So, once we had rounded off our work on skeletal muscle, Denis and I turned to explore the role of chloride in cardiac muscle. We used Purkinje fibers from sheep or dog hearts which mostly showed spontaneous rhythmic activity. Here are the main findings of our paper (Hutter and Noble 1959, 1961). Edward Carmeliet (1961) from Silvio Weidmann's laboratory reached essentially the same conclusions independently in his paper.

(1) Replacement of chloride by methylsulphate produced only a small increase in membrane resistance, indicating that the contribution of chloride to the total membrane conductance is much smaller in cardiac than it is in skeletal muscle. (2) In spontaneously beating preparations, replacement of chloride by methylsulphate eventually slows the rhythm. This suggests that E_Cl_ is normally positive to the maximum diastolic potential, as would be expected if chloride was passively distributed according to the mean membrane potential over time. (3) Replacement of chloride by I or NO_3_, causes hyperpolarization and arrest or slowing of the rhythm. (4) On basis of the above measurements of bi‐ionic potentials and of measurements of membrane resistance, the relative permeability of anions was found to be: I >NO_3_ > Br >Cl.

So, the differences between skeletal and cardiac muscle were quite striking. In skeletal muscle, the combination of a high chloride conductance with a chloride equilibrium potential normally near the resting potential serves to stabilize the resting potential. However, in spontaneously beating cardiac muscle fibers, the more positive value of the chloride equilibrium potential means that chloride current facilitates pacemaker activity, although the chloride conductance is relatively low. Most surprising to me of all was that the rank order of foreign anion permeability in cardiac muscle was the reverse of that in skeletal muscle.

I took up this theme again when Walmor Carlos deMello from Puerto Rico came to Mill Hill as a visiting worker and introduced us to crustacean muscle. Table 1 summarizes how we found the relative anion permeabilities to differ with muscle type and pH.

**Table 1:**
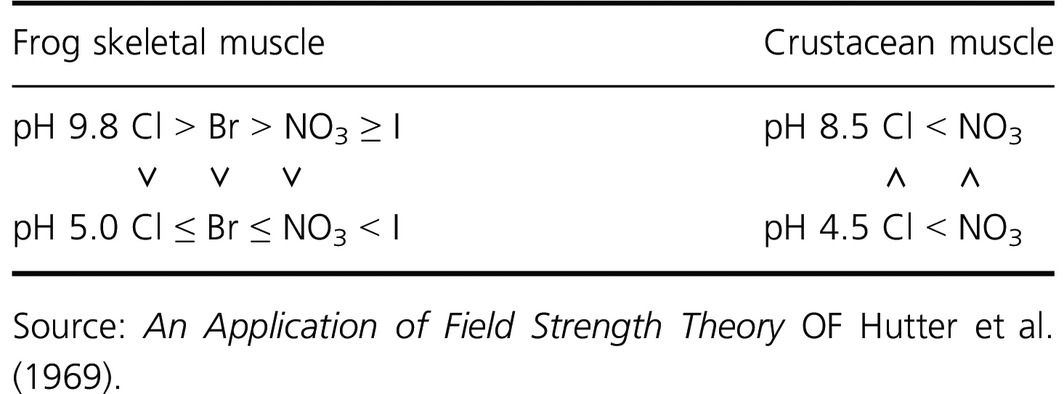
Relative anion permeabilities

Taking first the now familiar situation in skeletal muscle, the chloride permeability is greater in alkaline and neutral solution than in acid solution. And when the chloride permeability is high, bromide, nitrate, and iodide are progressively less permeant than chloride. But at pH 5, when the chloride permeability is low, the foreign anions – in particular iodide – are more permeable than is chloride (Hutter and Warner 1967d). In crustacean muscle by contrast, the chloride permeability is higher in acid solution than in alkaline solution. And throughout the pH range, nitrate is more permeable than is chloride.

Now at first sight, all this was perplexing. But it falls into place within the Field Strength theory developed by Eisenman (1961) to account for ionic selectivity. According to Eisenman, the relative tendency of an ion to enter into association with a channel site depends on the balance of two energies: the strength of the interaction of an ion with its hydration shell on the one hand, and on the other the strength of its interaction with the membrane site. If one then supposes that field strength of a membrane site with anion affinity can be increased by protonation, and that intrinsic field strength of such sites varies with species and muscle type, then such results can be rationalized. The difference between the anion selectivity of skeletal and cardiac muscle can similarly be accommodated.

Moving on now into the 1970s and 80s, the invention of chloride‐sensitive microelectrodes by J L Walker greatly advanced our knowledge of active chloride transport in the hands of several teams. As regards skeletal muscle, Bolton and Vaughan‐Jones (1977) confirmed the existence of active inward transport of chloride that is normally shunted by passive chloride movements. And later on Aickin et al. (1989) showed that a NaKCC co‐transport system was responsible for the inward transport of chloride.

As regards cardiac muscle, Richard Vaughan‐Jones (1979, 1986) found that even resting Purkinje fibers contained more chloride than would be expected if chloride were passively distributed as result of the operation of a chloride‐bicarbonate exchange mechanism. According to a more recent reviews (Vaughan‐Jones 2009; Alvarez‐Leefman 2012), an electrogenic chloride/bicarbonate transporter also serves as a mechanism for uphill chloride accumulation in cardiac cells, and this is further complemented by an NKCC transporter (Clemo et al. 1992) as in skeletal muscle and smooth muscle. In beating cardiac fibers, these mechanisms presumably work in addition to the effect the relatively low mean membrane potential has on the chloride distribution. Be that as it may, in cardiac muscle the chloride conductance – because of its relatively low value – normally continues to play second fiddle to the cations; though under pathological conditions, the potentially destabilizing effect of the chloride distribution may become arrhythmogenic (Huang and Duan 2011).

Moving now into the 1980s, two new techniques took the field. The first was the incorporation of membrane vesicles bearing ion channels into planar phospholipid bilayer systems. The second newcomer was the patch‐clamp technique. In the hands of Christopher Miller and his colleagues (White and Miller 1979; Miller and White 1984), these two approaches led to the discovery of a novel chloride channel in the electric organ of *Torpedo marmorata*. Thomas Jentsch and his colleagues then succeeded in cloning the electroplax chloride channel and this opened the way to the discovery of a whole family of chloride channels. Thus, Pusch et al (1991) identified and characterized the major skeletal muscle chloride channel which they called CLC‐1. It turned out that the single channel conductance of CLC‐1 is very low, only just 1pS. And this explains why attempts to find a convincing chloride channel through patch clamping of intact muscle fibers proved largely fruitless during the 1980s.

Now, it is neither possible nor necessary for me to describe how much the application of Molecular Biology has advanced our knowledge of anion permeability. A year ago, a special issue of *JPhysiol* celebrated 25 years of wonderfully productive research into chloride channels. Nevertheless, let me single out one remarkable finding that is especially cogent in present context. According to Fahlke et al. (1997), the pore properties of a recombinant human ClC‐1 channel are dramatically altered by substitution of Gly230 by glutamic acid (G230E). Although the anion selectivity of the wild‐type channel accords with our original findings on intact muscle fibers with chloride as the most permeable anion, the G230E variant exhibits the reverse selectivity series with chloride as the least permeable anion of the lyotropic series. Moreover, the cation to anion permeability ratio of the mutant channel is much greater than that of the wild‐type channel, suggesting that the anion conductance of fibers expressing the mutant channel would be abnormally low for skeletal muscle. So effectively, that single substitution of glycine by glutamate would result in a skeletal muscle fiber that mimics the properties of cardiac muscle as regards to anion permeability. Could then a naturally occurring glutamic acid residue at a critical site be the cause of the observed differences between the anion conductances of skeletal and cardiac muscle?

The broad picture, as far as I can see it, is that great advances have been made regarding the molecular architecture of chloride channels, and in regard to their equally novel gating mechanism. As regards ionic selectivity, however, the principles formulated now 60 years ago by George Eisenman have continued to find application. If I have one small quibble, then it is that in reworking the properties of chloride channels in expression systems, earlier relevant work has often been overlooked, as if it belonged to a closed dark age before the dawn of molecular biology.

Finally, while much of the modern work on chloride channels has been done by use of in vitro expression systems, our knowledge of the role of chloride in the functioning of skeletal muscle has also taken a new turn. From the beginning it was obvious that the high chloride conductance of skeletal muscle exerted a stabilizing influence on its excitability. And this function was driven home by Adrian and Bryant's (1974) demonstration that a low chloride conductance was the cause of the involuntary repetitive discharge characteristic of myotonia. But, in the last decade or so, an astonishing new twist has been given to the physiological role of chloride in controlling muscle excitability. The Danish team led by Thomas Pedersen has shown that during normal muscle action potential activity, multiple cellular signals control the chloride conductance. With the onset of activity the chloride channels are inhibited, which serves to preserve excitability in the face of potassium accumulation within the transverse tubules. But during prolonged activity that inhibition is lifted and the chloride conductance starts to rise. This late effect reduces the excitability of metabolically exhausted muscle fibers and so protects them from suffering damage.

All this and more is covered in the splendid, recent review by Pedersen et al. (2016). I found reading that admirably lucid review more enthralling than the most exciting thriller. And I hope that in good time you also will experience that exquisite pleasure which is reserved for those whose laboratory days are long behind them, but who can still take delight in how physiology is flourishing.

## Conflict of Interest

None declared.

## References

[phy213165-bib-0001] Adrian, R. H. 1961 Internal chloride concentration and chloride efflux of frog muscle. J. Physiol. 151:154–185. 10.1113/jphysiol.1961.sp006698 PMC1359908 13681533

[phy213165-bib-0002] Adrian, R. H. , and S. H. Bryant . 1974 On the repetitive discharge in myotonic muscle. J. Physiol. 240:505–515. 4420758 10.1113/jphysiol.1974.sp010620 PMC1331026

[phy213165-bib-0003] Adrian, R. H. , W. H. Chandler , and A. L. Hodgkin . 1970 Voltage clamp experiments in striated muscle fibres. J. Physiol. 208:607–644. 5499787 10.1113/jphysiol.1970.sp009139 PMC1348789

[phy213165-bib-0004] Aickin, C. C. , W. J. Betz , and G. L. Harris . 1989 Intracellular chloride and the mechanism for its accumulation in rat lumbrical muscle. J. Physiol. 411:437–455. 2515275 10.1113/jphysiol.1989.sp017582 PMC1190533

[phy213165-bib-0005] Alvarez‐Leefman, F. J . (2012).Chapter15 in Cell Physiology Source Book: essentials of Biophysics. Pp. 221–259 in Sperakelis Nicholas eds. Academic Press, San Diego.

[phy213165-bib-0006] Bolton, T. B. , and R. D. Vaughan‐Jones . 1977 Continuous direct measurement of intracellular chloride and pH in frog skeletal muscle. J. Physiol. 270:801–833. 20501 10.1113/jphysiol.1977.sp011983 PMC1353546

[phy213165-bib-0007] Boyle, P. J. , and E. J. Conway . 1941 Potassium accumulation in muscle and associated changes. J. Physiol. 100:1–63. 16991506 10.1113/jphysiol.1941.sp003922 PMC1393293

[phy213165-bib-0008] Boyle, F. J. , E. J. Conway , F. Kane , and H. L. O'Reilly . 1941 Volume of interfibre spaces in frog muscle and the calculation of concentrations in the fibre water. J. Physiol. 99:401–414. 16995261 10.1113/jphysiol.1941.sp003911 PMC1394100

[phy213165-bib-0009] Carmeliet, E. E. 1961 Chloride ions and the membrane potential of Purkinje fibres. J. Physiol. 156:375–388. 13690854 10.1113/jphysiol.1961.sp006682 PMC1359892

[phy213165-bib-0010] Clemo, H. F. , J. J. Feher , and C. M. Baumgarten . 1992 Modulation of rabbit ventricular cell volume and NKCC ‐cotransport by cGMP and atrial natriuretic factor. J. Gen. Physiol. 100:89–114. 1355106 10.1085/jgp.100.1.89 PMC2229121

[phy213165-bib-0011] Conway, E. J. , and P. J. Boyle . 1939 A mechanism for the concentration of potassium by cells, with experimental verification for muscle. Nature, Lond. 144:709.

[phy213165-bib-0012] Donnan, F. G. 1911 Theorie der Membrangleichgewichte und Membranpotentiale bei Vorhandensein von nicht dialysierenden Electrolyten. Ein Beitrag zur physikalisch‐chemieschen Physiology. Zeitschrift fur Elektrochemie und angewandte pysikalische Chemie 17:572–581.

[phy213165-bib-0013] Eisenman, G. 1961 On the elementary atomic origin of equilibrium ionic specificity in Kleinzeller A. and Kotyuk A., eds. Symposium on Membrane Transport and Metabolism. Academic Press, New York.

[phy213165-bib-0014] Fahlke, C. , C. L. Beck , and A. L. George . 1997 A mutation in autosomal dominant myotonia congenital affects pore properties of the muscle chloride channel. Proc. Natl Acad. Sci. USA 94:2729–2734. 9122265 10.1073/pnas.94.6.2729 PMC20158

[phy213165-bib-0015] Fenn, W. O. 1936 Electrolytes in Muscle. Physiol. Rev. 16:450–487.

[phy213165-bib-0016] Fenn, W. O. , D. M. Cobb , and B. S. March . 1934 Sodium and chloride in frog muscle. Amer. J. Physiol. 110:261–272.

[phy213165-bib-0017] Harris, E. J. 1958 Anion interaction in frog muscle. J. Physiol. 141:351–365. 13539844 10.1113/jphysiol.1958.sp005979 PMC1358806

[phy213165-bib-0018] Hill, A. V. , and L. McPherson . 1954 The influence of nitrate, iodide and bromide on the duration of the active state.in skeletal muscle. Proc. Roy. Soc. B 143:81–102. 13224652 10.1098/rspb.1954.0055

[phy213165-bib-0019] Höber, R. 1905 Uber den Einfluss der Salze auf den Ruhestrom des Froschmuskels. Pflügers Arch. 106:599–635.

[phy213165-bib-0020] Hodgkin, A. L. , and P. Horowicz .1957 Effect of potassium and chloride on the membrane potential of isolated muscle fibres. J. Physiol.. 137:30. 13439608

[phy213165-bib-0021] Hodgkin, A. L. , and P. Horowicz . 1959 The influence of potassium and chloride ions on the membrane potential of single muscle fibres. J. Physiol. 148:127–160. 14402240 10.1113/jphysiol.1959.sp006278 PMC1363113

[phy213165-bib-0022] Huang, Z. M. , and D. D. Duan . 2011 The functional role of chloride channels in cardiac pacemaker activity Pp. 573–594 in: Das M. R., eds. Modern pacemakers –present and future. InTech, Croatia.

[phy213165-bib-0023] Hutter, O. F. 2006 Some deconstructed dogmas. Prog. Biophys. Mol. Biol. 90:5–12. 16150479 10.1016/j.pbiomolbio.2005.06.002

[phy213165-bib-0024] Hutter, O. F. , and D. Noble . 1959 The influence of anions on impulse generation and membrane conductance in Purkinje and myocardial fibres. J. Physiol. 147:16–17P.

[phy213165-bib-0025] Hutter, O. F. , and D. Noble . 1960 The chloride conductance of frog skeletal muscle. J. Physiol. 151:89–102. 14405647 PMC1363222

[phy213165-bib-0026] Hutter, O. F. , and D. Noble . 1961 Anion conductance of cardiac muscle. J. Physiol. 157:335–350. 13717087 10.1113/jphysiol.1961.sp006726 PMC1359956

[phy213165-bib-0027] Hutter, O. F. , and S. M. Padsha . 1956 Effect of nitrate on the electrotonic potential of muscle. J. Physiol. 132:32P.13320418

[phy213165-bib-0028] Hutter, O. F. , and S. M. Padsha . 1959 Effect of nitrate and other anions on the membrane resistance of frog skeletal muscle. J. Physiol. 146:117–132. 13655220 10.1113/jphysiol.1959.sp006182 PMC1356894

[phy213165-bib-0029] Hutter, O. F. , and W. Trautwein . (1956). Vagal and sympathetic effects on the pacemaker fibres in the sinus venosus of the heart. J.gen.Physiol 39:715–733. 13319658 10.1085/jgp.39.5.715 PMC2147564

[phy213165-bib-0030] Hutter, O. F. , and A. E. Warner . 1967a The pH sensitivity of the chloride conductance of frog skeletal muscle. J. Physiol. 189:403–425. 6040154 10.1113/jphysiol.1967.sp008176 PMC1396114

[phy213165-bib-0031] Hutter, O. F. , and A. E. Warner . 1967b The effect of pH on the 36Cl efflux fromfrom frog skeletal muscle. J. Physiol. 189:427–443. 6040155 10.1113/jphysiol.1967.sp008177 PMC1396123

[phy213165-bib-0032] Hutter, O. F. , and A. E. Warner . 1967c Action of some foreign cations and anions on the chloride permeability of frog skeletal muscle. J. Physiol. 189:445–460. 6040156 10.1113/jphysiol.1967.sp008178 PMC1396121

[phy213165-bib-0033] Hutter, O. F. , and A. E. Warner . 1967d The anion discrimination of the skeletal muscle membrane. J. Physiol. 194:61–62P. 5639373

[phy213165-bib-0034] Hutter, O. F. , and A. E. Warner . 1972 The voltage dependence of the chloride conductance of frog muscle. J. Physiol. 227:275–290. 4539587 10.1113/jphysiol.1972.sp010032 PMC1331275

[phy213165-bib-0035] Hutter, O. F. , W. C. Mello , and A. E. Warner . 1969 An application of the field strength theory Pp. 391–400 in Tosteson D. C., eds. The Molecular Basis of Membrane Function. Prentice‐Hall, Englewood Cliffs, New Jersey.

[phy213165-bib-0036] Michaelis, L. 1925 Contribution to the theory of permeability of membranes for electrolytes. J. Gen. Physiol. 8:33–59. 19872189 10.1085/jgp.8.2.33 PMC2140746

[phy213165-bib-0037] Miller, C. , and M. M. White . 1984 Dimeric structure of Cl‐channels from Torpedo electroplax. Proc. Natl Acad. Sci. USA 81:2772–2775. 6326143 10.1073/pnas.81.9.2772 PMC345152

[phy213165-bib-0038] Mond, R. , and K. Amson . 1928 Uber die Ionenpermeabilitat des quergestreiften Muskels. Pflugers. Archiv. 220:69–81.

[phy213165-bib-0039] Padsha, S.M . 1957 The influence of anions on the membrane potential of skeletal muscle. J.Physiol 137:26,P. 13439606

[phy213165-bib-0040] Pedersen, T. H. , A. Risager , F. Vicenza de Paoli , T.‐Y. Chen , and O. B. Nielsen . 2016 Role of physiological CLC‐1 Cl ion channel regulation for the excitability and function of working skeletal muscle. J. Gen. Physiol. 147:291–308. 27022190 10.1085/jgp.201611582 PMC4810071

[phy213165-bib-0041] Popper, K. R. 1963 Conjectures and refutations: the growth of scientific knowledge. Routledge, London.

[phy213165-bib-0042] Pusch, M. , K. Steinmeyer , and T. J. Jentsch . 1991 Low single channel conductance of the major skeletal muscle chloride channel, CLC‐1. Biophys. J . 66:149–152. 10.1016/S0006-3495(94)80753-2 PMC1275674 8130334

[phy213165-bib-0043] Vaughan‐Jones, R. D. 1979 Non‐passive chloride distribution in mammalian heart muscle: micro‐electrode measurement of the intracellular chloride activity. J. Physiol. 295:83–109. 521996 10.1113/jphysiol.1979.sp012956 PMC1278788

[phy213165-bib-0044] Vaughan‐Jones, R. D. 1986 An investigation of chloride‐bicarbonate exchange in the sheep cardiac Purkinje fibre. J. Physiol. 379:377–406. 3559998 10.1113/jphysiol.1986.sp016259 PMC1182903

[phy213165-bib-0045] Vaughan‐Jones, R. D. 2009 Intracellular pH regulation in the heart. J. Moll. Cell. Cardiol. 46:318–331. 10.1016/j.yjmcc.2008.10.024 19041875

[phy213165-bib-0046] Warner, A. E. 1972 Kinetic properties of the chloride conductance of frog muscle. J. Physiol. 227:291–313. 4539588 10.1113/jphysiol.1972.sp010033 PMC1331276

[phy213165-bib-0047] White, M. M. , and C. Miller . 1979 A voltage–gated anion channel from the electric organ of *Torpedo californica* . J. Biol. Chem. 254:10161–10166. 489590

